# SABINA + Hong Kong: a territory wide study of prescribing trends and outcomes associated with the use of short-acting β2 agonists in the Chinese population

**DOI:** 10.1186/s12890-024-03038-1

**Published:** 2024-05-14

**Authors:** Lydia WY Fung, Vincent KC Yan, Christine Kwan, WC Kwok, David CL Lam, Christine F McDonald, Chloe I Bloom, Ian CK Wong, Esther W Chan

**Affiliations:** 1 Centre for Safe Medication Practice and Research, Department of Pharmacology and Pharmacy, Li Ka Shing Faculty of Medicine, The University of Hong Kong, Hong Kong SAR, China; 2 Laboratory of Data Discovery for Health (D24H), Hong Kong Science and Technology Park, Hong Kong SAR, China; 3 Sau Po Centre of Ageing, The University of Hong Kong, Hong Kong SAR, China; 4 Department of Medicine, School of Clinical Medicine, Li Ka Shing Faculty of Medicine, Queen Mary Hospital, The University of Hong Kong, Hong Kong SAR, China; 5 Department of Respiratory and Sleep Medicine, Austin Health, Melbourne, Australia; 6 National Heart and Lung Institute, Imperial College London, London, UK; 7 Aston Pharmacy School, Aston University, Birmingham, B4 7ET UK; 8 Department of Pharmacy, The University of Hong Kong-Shenzhen Hospital, Shenzhen, China; 9 The University of Hong Kong Shenzhen Institute of Research and Innovation, Shenzhen, China; 10 formerly, Centre for Safe Medication Practice and Research, Department of Pharmacology and Pharmacy, Li Ka Shing Faculty of Medicine, The University of Hong Kong, Hong Kong SAR, China; 11 University of Melbourne, Parkville, Melbourne, Victoria Australia; 12 Institute for Breathing and Sleep, Heidelberg, Melbourne, Victoria Australia; 13 Advanced Data Analytics for Medical Science (ADAMS) Limited, Hong Kong SAR, China; 14 formerly, Laboratory of Data Discovery for Health (D24H), Hong Kong Science and Technology Park, Hong Kong SAR, China

**Keywords:** Asthma, Short-acting β2 agonist, Mortality, Chinese population

## Abstract

**Background:**

Excessive use of short-acting β2 agonists (SABA) in patients with asthma continues to be a notable concern due to its link to higher mortality rates. Global relevance of SABA overuse in asthma management cannot be understated, it poses significant health risk to patients with asthma and imposes burden on healthcare systems. This study, as part of global SABINA progamme, aimed to describe the prescribing patterns and clinical outcomes associated with SABA use in the Chinese population.

**Methods:**

Retrospective cohort study was conducted using anonymized electronic healthcare records of Clinical Data Analysis and Reporting System (CDARS) from Hong Kong Hospital Authority (HA). Patients newly diagnosed with asthma between 2011 and 2018 and aged ≥12 years were included, stratified by SABA use (≤2, 3–6, 7–10, or ≥11 canisters/year) during one-year baseline period since asthma diagnosis date. Patients were followed up from one-year post-index until earliest censoring of events: outcome occurrence and end of study period (31 December 2020). Cox proportional regression and negative binomial regression were used to estimate the mortality risk and frequency of hospital admissions associated with SABA use respectively, after adjusting for age, sex, Charlson Comorbidity Index (CCI), and inhaled corticosteroid (ICS) dose. Outcomes include all-cause, asthma-related, and respiratory-related mortality, frequency of hospital admissions for any cause, and frequency of hospital admissions due to asthma.

**Results:**

17,782 patients with asthma (mean age 46.7 years, 40.8% male) were included and 59.1% of patients were overusing SABA (≥ 3 canisters per year). Each patient was prescribed a median of 5.61 SABA canisters/year. SABA overuse during baseline period was associated with higher all-cause mortality risk compared to patients with ≤2 canisters/year. Association was dose-dependent, highest risk in those used ≥11 canisters/year (adjusted hazard ratio: 1.42, 95% CI: 1.13, 1.79) and 3–6 canisters/year (adjusted hazard ratio: 1.22, 95% CI: 1.00, 1.50). Higher SABA prescription volume associated with increased frequency of hospital admissions with greatest risk observed in 7–10 canisters/year subgroup (adjusted rate ratio: 4.81, 95% CI: 3.66, 6.37).

**Conclusions:**

SABA overuse is prevalent and is associated with increased all-cause mortality risk and frequency of hospital admissions among the patients with asthma in Hong Kong.

**Supplementary Information:**

The online version contains supplementary material available at 10.1186/s12890-024-03038-1.

## Introduction

Asthma is the most common chronic respiratory diseases that affects people of all ages, with a global prevalence of 262 million in 2019 [1]. In Hong Kong, about 68,000 persons were diagnosed with asthma in 2019, accounting for 1% of the total population [2]. Asthma symptoms range from mild coughing and wheezing to life-threatening exacerbation. The goals of asthma treatment are to achieve symptomatic control, minimize the risk of acute exacerbation, and minimize treatment toxicity.

For the last several decades, the use of short-acting β2 agonists (SABA) alone as an intermittent reliever medication (step 1) or with the additional use of a controller medication, low-dose inhaled corticosteroid (ICS) (step 2), have been recommended for the treatment of patients with mild asthma [3, 4]. Due to the minor and occasional nature of symptoms in mild asthma, many patients rely on SABA alone to relieve symptoms, with poor adherence to regular ICS that addresses the underlying inflammatory pathology of asthma [5], leading to an increased risk of severe asthma exacerbations [6]. Evidence shows that excessive use of SABA (≥11 canisters per year), as monotherapy or in combination with ICS, is associated with an increased risk of asthma-related mortality [7, 8]. Since patients with mild asthma account for 50-75% of the asthma population [9], over-reliance on SABA has been a cause for concern. In 2019, to reduce the risk of severe exacerbations in people with mild asthma, the Global Initiative for Asthma (GINA) reported that SABA alone without ICS was no longer recommended. Instead, all adults and adolescents with asthma are recommended to use ICS-containing controller treatment for symptomatic relief (steps 1 to 2) or daily use (steps 2 to 5). This heralds a paradigm shift in asthma management [10]. 

The importance of addressing SABA overuse in asthma is significant on a global scale. The SABINA (SABA use IN Asthma) Program is a global research program that aims to describe and understand the treatment pattern of asthma medications, the extent of SABA inhaler use and the associations between SABA use and different clinical outcomes in different parts of the world [11]. SABINA Europe reported excessive use of SABA and poor adherence to ICS among patients with asthma. Overuse of SABA was found to range from 9% in Italy to 38% in the United Kingdom [12]. The SABINA Sweden cohort study with 365,324 patients showed that the risk of exacerbation and mortality rose with increased SABA use. Patients who used ≥11 canisters per year had a two-fold risk of death compared to those who received ≤2 canisters per year [13]. Although there are quite a few studies on the topic, data from an Asian population is scarce. A population-based study in Korea showed that the rate of SABA overuse was about 2–4% among patients with asthma [14]. Except for a recent SABINA study in Taiwan that showed a prevalence rate of 15.9% of SABA overuse and an association between SABA overuse and increased risk of severe exacerbation and all-cause mortality [15]. Studies conducted in various countries have demonstrated a link between SABA overuse and adverse outcomes in patients with asthma. However, data on the treatment pattern of asthma and the clinical outcomes associated with SABA use in the Chinese population are limited. This study, as part of the global SABINA progamme, aimed to describe the prescribing patterns and clinical outcomes associated with SABA use in the Hong Kong population.

## Methodology

### Data source

This was a retrospective population-based cohort study using anonymized electronic healthcare records of the Clinical Data Analysis and Reporting System (CDARS) from the Hong Kong Hospital Authority (HA). The HA serves all residents in Hong Kong (over 7 million), covering approximately 80% of all hospital admissions and providing ongoing medical treatment for 76% of patients with chronic health conditions through 43 hospitals and institutions [16, 17], 49 specialist outpatient clinics, and 73 general outpatient clinics. Several high-quality pharmaco-epidemiological studies have used CDARS data in the past [16–20]. Data validity and reliability of the database are reflected by the high coding accuracy for clinical outcomes as reported in previous studies with high positive and negative predictive values of more than 90% [16, 18, 20]. Therefore, CDARS is a nationwide source of medical records covering outpatient and inpatient healthcare records as well as mortality data, representative of the population in Hong Kong.

### Study design

**Fig. 1:**
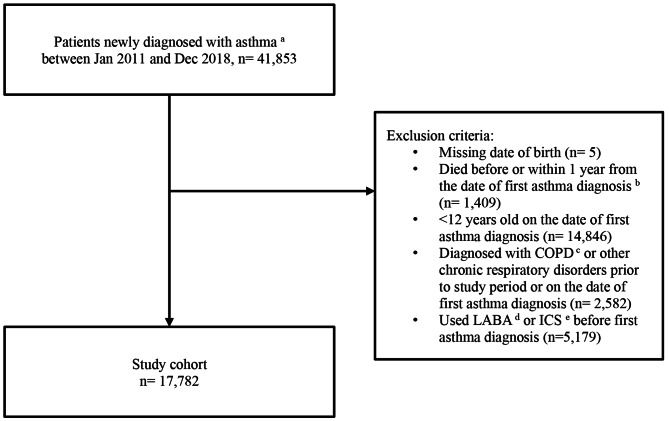
Flowchart of cohort identification ^a^ Date of admission would be considered as the index date if the index diagnosis is an inpatient episode ^b^ Removal of patients who died within 1 year from the date of first asthma diagnosis due to insufficient baseline period to ascertain SABA use ^c^ COPD = chronic obstructive pulmonary disease ^d^ LABA = long acting ß2 agonist ^e^ ICS = inhaled corticosteroid

Patients diagnosed with asthma and aged ≥12 years between January 1, 2011 and December 31, 2018 were identified using the International Classification of Diseases–9th Edition (ICD-9) code 493.x. Patients with a history of chronic obstructive pulmonary disease or a chronic respiratory disease other than asthma on or before the date of first asthma diagnosis, those who received long-acting β2 agonists (LABA) and/or ICS prescription before first asthma diagnosis, or those who died on or within one year from the date of study entry were excluded. The index date was defined as the date of first asthma diagnosis. The baseline period starts from the index date up to one-year post-index, during which patients were categorized based on SABA use (≤2, 3–6, 7–10, or ≥11 canisters per year). Patients were followed up from one-year post-index until the earliest censoring of events: occurrences of outcome(s), end of the study period December 31,2020) or death (Fig. 1).

### Outcomes, other variables, and covariates

Outcomes include all-cause mortality, asthma-related mortality (defined as the cause of death with ICD-10 code J45), respiratory-related mortality (defined as the cause of death with ICD-10 code J00-J99), frequency of hospital admissions for any cause, and frequency of hospital admissions due to asthma (defined as hospital admissions with a primary diagnosis of ICD-9 code 493.x). Covariates, including patient demographics (age, sex, year of first asthma diagnosis) at index date, health status (Charlson Comorbidity Index [CCI], hospitalization one year before the index date), pre-existing comorbidities (allergic rhinitis, gastroesophageal reflux disease, coronary artery disease, hypertension, diabetes, congestive heart failure, atrial fibrillation, stroke, renal disease, and cancer) before the index date, were described. Prescribing patterns and choice of asthma treatment during the baseline period (including the use of SABA, LABA, ICS, long-acting muscarinic antagonists [LAMA], leukotriene receptor antagonists [LTRA], anti-IgE / anti-IL5/5R / anti-IL4R, and oral corticosteroid [OCS] use) were reported. Asthma severity was assessed by the dose of ICS used during the baseline period, categorized into none, low, medium, or high with reference to the Global Strategy for Asthma Management and Prevention issued by GINA [10], and adjusted in the analyses.

### Statistical analysis

Patient characteristics, including covariates at baseline and choice of asthma treatment during the baseline period, were reported descriptively as frequencies (percentages) for categorical variables and mean (SD) for continuous variables. The proportion of patients receiving a prescription for SABA and the number of canisters prescribed per year during the baseline period were estimated. Patients were stratified by SABA use (≤2, 3–6, 7–10, or ≥11 canisters per year). SABA overuse was defined as patients who were prescribed ≥ 3 SABA canisters per year. Baseline characteristics for each subgroup were reported. The dose of ICS (low, medium, or high) and the use of other asthma treatments during the baseline period were described. The trend in SABA use (number of canisters per year per patient) in each calendar year during the study period was reported. Incidence rates of all outcomes were reported. Risks of all-cause mortality, respiratory-related deaths, and asthma-related deaths associated with SABA use were estimated using Cox proportional hazards regression, after adjusting for age, sex, CCI, and ICS dose. ICS dose was adjusted as a covariate in the analysis since asthma severity is associated with the risk of mortality. Hazard ratios with their 95% confidence intervals were reported. The frequency of hospital admissions associated with SABA use was estimated using negative binomial regression, after adjusting for age, sex, CCI, and ICS dose. Rate ratios and their 95% confidence intervals were reported. A p-value less than 0.05 was considered statistically significant in all analyzes. All analysis was performed using R 4.0.5 (R Foundation for Statistical Computing, Vienna, Austria) and cross-checked by two independent investigators (LF and VY).

## Results

After applying the exclusion criteria, we included a total of 17,782 patients with a diagnosis of asthma between January 1, 2011 to December 31, 2018 (Fig. 1). The mean age was 46.7 years and 40.8% were men. (Table 1). The majority of patients had no comorbidities (81.9%) or a very mild comorbidity score (15.1%). Major comorbidities among the study cohort were hypertension (9.5%) and diabetes (4.3%) respectively. Patients receiving more SABA canisters per year during the baseline period were generally older, had more comorbidities, and were more likely to have severe asthma as reflected by the ICS dose and the use of LAMA, LTRA, and ICS.

**Table 1:** Baseline characteristics of users of short-acting β2-agonists (SABA) during baseline period

		Number of SABA canisters prescribed during baseline period			
Baseline characteristics ^a^	Overall	≤2	3–6	7–10	≥11
**N**	17,782	7,266 (40.9)	3,276 (18.4)	1,846 (10.4)	5,394 (30.3)
**Demographics**					
Sex, male	7,257(40.8)	3,005 (41.4)	1,318 (40.2)	744 (40.3)	2,190 (40.6)
Age, mean (SD), y	46.66 (20.89)	39.66 (17.96)	44.54 (20.27)	47.20 (20.33)	57.18 (20.82)
**Charlson Comorbidity Index** ^**b**^					
0	14,561 (81.9)	6,519 (89.7)	2,769 (84.5)	1,491 (80.8)	3,782 (70.1)
1–2	2,678 (15.1)	656 (9.0)	433 (13.2)	314 (17.0)	1,275 (23.6)
3–4	421 (2.4)	67 (0.9)	48 (1.5)	34 (1.8)	272 (5.0)
≥5	122 (0.7)	24 (0.3)	26 (0.8)	7 (0.4)	65 (1.2)
**Hospitalization 1 year prior to asthma diagnosis**					
Number of hospitalizations mean (SD)	0.41 (1.46)	0.31 (1.06)	0.32 (1.14)	0.42 (1.60)	0.59 (1.95)
**Comorbidities prior to asthma diagnosis**					
Allergic rhinitis	306 (1.7)	121 (1.7)	61 (1.9)	38 (2.1)	86 (1.6)
Gastroesophageal reflux disease	139 (0.8)	45 (0.6)	21 (0.6)	12 (0.7)	61 (1.1)
Coronary artery disease	604 (3.4)	122 (1.7)	78 (2.4)	51 (2.8)	353 (6.5)
Hypertension	1,685 (9.5)	335 (4.6)	224 (6.8)	173 (9.4)	953 (17.7)
Diabetes	758 (4.3)	176 (2.4)	103 (3.1)	65 (3.5)	414 (7.7)
Congestive heart failure	531 (3.0)	60 (0.8)	56 (1.7)	39 (2.1)	376 (7.0)
Atrial fibrillation	375 (2.1)	59 (0.8)	58 (1.8)	38 (2.1)	220 (4.1)
Stroke	344 (1.9)	75 (1.0)	46 (1.4)	30 (1.6)	193 (3.6)
Renal disease	317 (1.8)	64 (0.9)	42 (1.3)	31 (1.7)	180 (3.3)
Cancer	426 (2.4)	98 (1.3)	74 (2.3)	53 (2.9)	201 (3.7)
	**Overall** **(** ***N*** **= 17,782)**	**≤2** **(** ***N*** **= 7,266)**	**3–6** **(** ***N*** **= 3,276)**	**7–10** **(** ***N*** **= 1,846)**	**≥11** **(** ***N*** **= 5,394)**
**ICS dose** ^**c**^					
None	10,066 (56.6)	6,799 (93.6)	1,860 (56.8)	564 (30.6)	843 (15.6)
Low	2,406 (13.5)	206 (2.8)	518 (15.8)	463 (25.1)	1,219 (22.6)
Medium	4,407 (24.8)	210 (2.9)	765 (23.4)	703 (38.1)	2,729 (50.6)
High	903 (5.1)	51 (0.7)	133 (4.1)	116 (6.3)	603 (11.2)
**LABA prescription** ^**d**^	3,072 (17.3)	268 (3.7)	431 (13.2)	384 (20.8)	1,989 (36.9)
No regular ICS prescription ^e^	167 (5.5)	74 (27.6)	64 (14.9)	14 (3.7)	15 (0.8)
With regular low-dose ICS	753 (24.5)	78 (29.1)	122 (28.3)	104 (27.1)	449 (22.6)
With regular medium-dose ICS	1,546 (50.3)	86 (32.1)	175 (40.6)	201 (52.3)	1,084 (54.5)
With regular high-dose ICS	606 (19.7)	30 (11.2)	70 (16.2)	65 (16.9)	441 (22.1)
**LAMA prescription** ^**f**^	396 (2.2)	15 (0.2)	19 (0.6)	21 (1.1)	341 (6.3)
**LTRA prescription** ^**g**^	853 (4.8)	85 (1.2)	101 (3.1)	99 (5.4)	568 (10.5)
**Anti-IgE, Anti-IL5/5R, Anti-IL4R prescription, n (%)**	1 (0.0)	0 (0.0)	0 (0.0)	0 (0.0)	1 (0.0)
**In-patient oral corticosteroid (OCS) use**	5,705 (32.1)	529 (7.3)	1,115 (34.0)	798 (43.2)	3,263 (60.5)

^a^Values are expressed as frequency (%) unless otherwise specified

^b^Charlson Comorbidity Index (CCI) indicates patients with myocardial infarction, congestive heart failure, peripheral heart disease, cerebrovascular disease, chronic obstructive pulmonary disease, dementia, paralysis, diabetes (with or without sequalae), chronic renal failure, liver disease (mild or moderate to severe), ulcers, rheumatism and other inflammatory polyarthropathies, acquired-immune deficiency syndrome, malignancy and metastatic solid tumor. Severity of comorbidity was categorized into 3 grades based on the score: mild with scores 1–2; moderate with scores 3–4 and severe with scores 5 or above, where higher score indicates a higher risk of mortality

^c^ICS = inhaled corticosteroid, ICS dose was classified according to the GINA guidelines 2019

^d^LABA = long-acting β2-agonist

^e^No regular prescription was defined as patient received no more than 28 days of ICS prescription during the baseline year, there were 12 patients did not receive ICS prescription throughout the baseline year

^f^LAMA = long-acting muscarinic antagonists

^g^LTRA = leukotriene receptor antagonists

### Prescription pattern of SABA and other asthma medications

Among the study cohort, 59.1% of patients were overusing SABA (≥ 3 canisters per year), of which 3,276 (18.4%) patients were prescribed 3–6 canisters, 1,846 (10.4%) patients were prescribed 7–10 canisters, and 5,394 (30.3%) patients were prescribed ≥11 canisters during the one-year baseline period. Throughout the study period, the median SABA canisters prescribed to each patient with asthma per year was 5.61 canisters (Table 2). The overall prescription rate of ICS and LABA was only 43.4% and 17.3%, respectively. Patients who were prescribed a higher number of SABA canisters had higher number of ICS and LABA prescriptions (Table 1). The highest prescription volume of OCS was found in the ≥11 canisters/year subgroup followed by ≤2 canisters/year subgroup. The overall use of other asthma medications (LAMA and LTRA) was relatively low among patients with asthma at 2.2% and 4.8% respectively. Patients prescribed a higher number of SABA canisters also had a high proportion of prescribed LAMA and LTRA.

**Table 2:** Trend of SABA prescription among asthma patients from 2011 to 2020

Year	Canisters prescribed	Number of active patients	Canisters per patient	Number of active patients by SABA canister groups			
				≤2 canisters/year	3–6 canisters/year	7–10 canisters/year	≥11 canisters/year
2011	14,636	2,811	5.21	1,535	595	270	411
2012	29,307	5,387	5.44	3,078	892	507	910
2013	41,978	7,770	5.40	4,621	1,155	679	1,315
2014	56,762	9,825	5.78	6,058	1,106	775	1,886
2015	69,990	11,650	6.01	7,256	1,090	828	2,476
2016	78,857	13,610	5.79	8,493	1,467	1,117	2,533
2017	90,806	15,428	5.89	9,678	1,582	1,263	2,905
2018	99,136	17,090	5.80	10,831	1,700	1,418	3,141
2019	90,969	16,868	5.39	11,079	1,457	1,286	3,046
2020	80,623	16,631	4.85	11,427	1,303	1,201	2,700

### Risk of mortality associated with SABA use

After adjusting for age, sex, CCI and ICS dose, patients who were overusing SABA (≥ 3 canisters/year) during the baseline period had a higher risk of all-cause mortality compared to patients with appropriate use (≤ 2 canisters/year). The association was dose-dependent, with the highest risk in those who used ≥ 11 canisters/year (adjusted HR: 1.84, 95% CI: 1.55, 2.19) followed by patients who used 7–10 canisters/year (adjusted HR: 1.42, 95% CI: 1.13, 1.79) and 3–6 canisters/year (adjusted HR: 1.22, 95% CI: 1.00, 1.50) (Table 3). Despite a similar association observed in the risk of respiratory-related (Table 4) and asthma-related mortality (Table 5), the associations were not statistically significant. Only patients who used ≥ 11 SABA canisters/year showed a statistically significant increased risk of respiratory-related mortality (adjusted HR: 1.86, 95% CI: 2.09, 17.86) and asthma-related mortality (adjusted HR: 19.1, 95% CI: 1.95, 187.3) respectively.

**Table 3:** Risk of all-cause mortality among SABA canister groups

SABA canister use	No. of patients/ no. of deaths	Incidence Rate per 1,000 person years	Crude HR (95% CI)	Adjusted HR (95% CI)	*P* values
≤2 canisters/year	7,266/234	5.95	1.0 (Ref)	1.0 (Ref)	
3–6 canisters/year	3,276/177	10.41	1.75 (1.44, 2.13)	1.22 (1.00, 1.50)	0.050***
7–10 canisters/year	1,846/122	13.25	2.23 (1.79, 2.77)	1.42 (1.13, 1.79)	0.003***
≥11 canisters/year	5,394/831	33.95	5.69 (4.92, 6.58)	1.84 (1.55, 2.19)	< 0.001***

*Adjusted for age, sex, Charlson Comorbidity Index, ICS dose

**Table 4:** Risk of respiratory-related mortality among SABA canister groups

SABA canister use	No. of patients/ no. of deaths	Incidence Rate per 1,000 person years	Crude HR (95% CI)	Adjusted HR (95% CI)	*P* values
≤2 canisters/year	7,266/89	2.26	1.0 (Ref)	1.0 (Ref)	
3–6 canisters/year	3,276/63	3.71	1.64 (1.19, 2.27)	1.09 (0.78, 1.52)	0.612
7–10 canisters/year	1,846/47	5.11	2.26 (1.59, 3.22)	1.39 (0.68, 10.94)	0.08
≥11 canisters/year	5,394/351	14.34	6.36 (5.04, 8.02)	1.86 (2.09, 17.86)	< 0.001***

*Adjusted for age, sex, Charlson Comorbidity Index, ICS dose

**Table 5:** Risk of asthma-related mortality among SABA canister groups

SABA canister use	No. of patients/ no. of deaths	Incidence Rate per 1,000 person years	Crude HR (95% CI)	Adjusted HR (95% CI)	*P* values
≤2 canisters/year	7,266/1	0.03	1.0 (Ref)	1.0 (Ref)	
3–6 canisters/year	3,276/3	0.18	6.89 (0.72, 66.22)	6.93 (0.69, 69.8)	0.100
7–10 canisters/year	1,846/1	0.11	4.20 (0.26, 67.22)	4.27 (0.24,76.1)	0.324
≥11 canisters/year	5,394/13	0.53	19.81 (2.59, 151.55)	19.1 (1.95, 187.3)	0.011***

*Adjusted for age, sex, Charlson Comorbidity Index, ICS dose

### Frequency of hospital admission associated with SABA use

After adjusting for age, sex, CCI, and ICS dose, an increased number of prescribed SABA canisters was associated with an increased frequency of hospital admissions, although a dose-response relationship was not observed (Table 6). The highest risk was observed in the 7–10 canisters/year subgroup (adjusted RR: 4.81, 95% CI: 3.66, 6.37) which was higher than the ≥ 11 canisters/year subgroup (adjusted RR: 3.72, 95% CI: 2.98, 4.66) and 3–6 canisters/year subgroup (adjusted RR: 2.74, 95% CI: 2.16, 3.49). On the other hand, the frequency of asthma-related hospital admission was only found to be statistically significant among ≥ 11 canisters/year subgroup (adjusted RR: 3.62, 95% CI: 2.27, 5.82) but not in the 3–6 canisters/year and 7–10 canisters/year subgroups. (Table 7).

**Table 6:** Frequency of hospital admissions associated with SABA use

SABA canister use	No. of patients/ no. of admissions	Crude RR (95% CI)	Adjusted RR (95% CI)	*P* values
≤2 canisters/year	7,266/2,240	1.0 (Ref)	1.0 (Ref)	
3–6 canisters/year	3,276/1,797	1.78 (1.39, 2.30)	2.74 (2.16, 3.49)	< 0.001***
7–10 canisters/year	1,846/1367	2.40 (1.79, 3.30)	4.81 (3.66, 6.37)	< 0.001***
≥11 canisters/year	5,394/8,453	5.08 (4.11, 6.30)	3.72 (2.98, 4.66)	< 0.001***

*Adjusted for age, sex, Charlson Comorbidity Index, ICS dose

**Table 7:** Frequency of asthma-related hospital admission associated with SABA use

SABA canister use	No. of patients/ no. of admissions	Crude RR (95% CI)	Adjusted RR (95% CI)	*P* values
≤2 canisters/year	7,266/61	1.0 (Ref)	1.0 (Ref)	
3–6 canisters/year	3,276/74	2.69 (1.72, 4.24)	1.52 (0.91, 2.56)	0.11
7–10 canisters/year	1,846/33	2.13 (1.22, 3.74)	1.12 (0.60, 2.07)	0.74
≥11 canisters/year	5,394/665	14.69 (10.26, 21.26)	3.62 (2.27, 5.82)	< 0.001***

*Adjusted for age, sex, Charlson Comorbidity Index, ICS dose

### Subgroup analysis

We conducted a subgroup analysis stratified by patients with OCS prescriptions (Supplementary Tables 1–2). Among patients with any OCS prescription during the baseline period, the risk of all-cause mortality was consistent with the main analysis and a dose-response relationship with the increase in SABA use was also observed. Statistically significant relationship was observed for the frequency of hospital admission among patients with OCS prescriptions, but a dose-response relationship was not observed.

## Discussion

In this Hong Kong-wide study, SABA overuse was observed in more than half (∼ 60%) of the study population. In particular, more than 30% of patients had 11 or more SABA canisters prescribed within their first year of asthma diagnosis. These results suggest that over-prescription of SABA and potential SABA overuse was considerably more serious in Hong Kong than in other countries such as Taiwan [15], Korea [14], Sweden [13], and other parts of Europe [12] which only had prevalence rates ranging from 16 to 30%.

Consistent with SABINA studies in Europe and Taiwan [12, 13, 15], SABA overuse in Hong Kong was associated with a statistically significant increased risk of all-cause mortality as well as frequency of hospital admissions, after adjusting for age, sex, health status, and asthma severity (in terms of ICS dose). The risk of all-cause mortality increased significantly with SABA overuse, even for those with mild overuse (3–6 canisters/year), and a dose-dependent trend was observed, which further consolidated the statistical association. Since SABA is the reliever medication in asthma treatment and does not possess anti-inflammation effects, high use of SABA for asthma management is merely symptom relieving and does not manage the underlying inflammation, suggesting suboptimal asthma management [21]. Importantly, this leads to progressive worsening of symptoms and other adverse events, which eventually increases the risk of all-cause mortality. The exact biological mechanism between SABA overuse and all-cause mortality is not completely understood, however all-cause mortality is considered as an important indicator for assessing the safety of long-term medications among asthma patients [22, 23]. Among the all-cause mortality events, the majority (40.3%) were respiratory-related, and other common causes (1.2-10.6%) include cancer (lung, liver or unspecified), heart failure, sepsis, acute myocardial infarction and chronic kidney disease.

A dose-dependent trend was not observed, however, for frequency of hospital admissions. Limited by low incidence rates of respiratory- and asthma-related mortality among the study population, no statistically significant association was observed between increased SABA use and risk of respiratory- and asthma-related mortalities, except in patients receiving ≥ 11 canisters per year. Future studies with a larger sample size would be needed to re-assess this potential association.

Despite the change to international guidelines in 2019 that as-needed SABA monotherapy was no longer recommended in patients with mild asthma and that such patients should receive ICS-containing controller treatment to reduce the risk of serious exacerbations and control symptoms [10], our data up to the end of 2020 revealed no evidence of a corresponding change in prescribing practice in Hong Kong. The overall prescription of LABA with ICS, which was the new asthma treatment recommendation, was low among patients with mild asthma. Therefore, physicians might be over-reliant on SABA as a reliever for patients with asthma and this might have contributed to poor symptomatic control and increased risk of adverse outcomes. Furthermore, the possibility of physicians over-prescribing SABA canisters to patients for stockpiling purposes could not be ruled out.

The findings of this study have clinical implications. Firstly, consistent with studies conducted in other countries, SABA overuse was associated with increased mortality and hospitalization even after accounting for age, sex, health status, and asthma severity. The research findings contribute to the global understanding of SABA overuse in asthma management particularly among the Chinese population, it reinforces the importance of addressing the issue not only in Hong Kong but also in other regions worldwide. Secondly, over-prescription of SABA to patients with asthma in Hong Kong was observed. Despite the change in recommendations to international guidelines, changes in local clinical practice to reduce SABA overuse were not evident in Hong Kong. It is imperative to identify the gaps and develop action plans for updating local clinical guidelines and changing clinical practice. The GINA treatment strategy is one of the main clinical guidelines used by physicians to assess asthma control [24]. Hence, promotion of changes in the GINA treatment strategy (as-needed low dose ICS-formoterol as the preferred controller and reliever option in steps 1–2 and removal of SABA monotherapy as the recommended reliever option) to physicians at clinics frequently attended by patients with mild asthma, such as General Out-patient Clinics (GOPC), Respiratory and Family Medicine Specialist Clinics, would be necessary. Currently, drug choices at GOPC are limited as patients are perceived to have mild disease [25], various ICS and combination medications such as ICS-formoterol are usually prescribed by respiratory specialists according to the Drug Formulary in HA, physicians at GOPC may tend to prescribe SABA as relievers to the patients among the limited asthma medication options, thus contributing to the SABA overuse. Thirdly, study findings have shed light on SABA over-prescription in clinical practice in Hong Kong and high prescription of OCS, indicating the need to critically review the standard drug formulary for treating asthma in primary care and specialty care clinics in local public health care settings, given that these are the contexts in which SABA over-prescription took place. For instance, a critical review of the drug formulary and prescribing practices for treating asthma in primary care and specialty care clinics should be warranted to minimize SABA overuse and adherence to controller medications. Access to ICS in primary care clinics should be considered a priority. Prescribing and dispensing practice should also be monitored over time to assess whether changes in drug availability would lead to the desired results and if additional factors should be considered including physician, patient, and/or systems. The study also highlights for increased awareness and education among healthcare providers regarding the appropriate use of SABA medications in asthma management. This includes understanding the potential risks associated with SABA overuse and the importance of promoting controller medications for long-term asthma control. Further investigations can look into the underlying factors contributing to SABA overuse such as patient preferences and healthcare system barriers which can help to develop a comprehensive understanding of SABA overuse in asthma management. Apart from the promotion of conventional treatment guidelines, the implementation of national or regional asthma programs [26] and encouraging patient involvement in disseminating appropriate treatment information [27] may also be effective measures to improve asthma care in the city. For instance, training primary care providers on the appropriate asthma management approach or enhance the access to specialized care, as well as establishment of robust data collection and surveillance systems to monitor asthma prevalence, control and medication use patterns. This can help identify areas of improvement and guide further adjustments in asthma management strategies. These strategies could also be useful in other regions worldwide.

### Limitations

Several limitations deserve attention. Firstly, prescription data were used to estimate SABA inhaler usage, hence reflecting only the number of SABA canisters dispensed to patients. Data on treatment adherence and stockpiling were not available; therefore, the actual consumption trend of patients with asthma may not be adequately reflected by the prescriptions and the actual use of SABA by patients might be overestimated. Secondly, only prescription data and patients treated in hospitals and clinics managed by the HA, the sole public health service provider in Hong Kong, were captured in this study, hence it may not be representative of the private healthcare sector. Lastly, as with any observational studies, the possibility of unmeasured residual confounding, such as socioeconomic status could not be ruled out. Such unmeasured confounding could potentially under- or over-estimate the risks associated with SABA overuse. Nevertheless, essential covariates associated with SABA use have been adjusted for in the main analysis, and the possibility of an unmeasured confounder with sufficient effect size to change our main conclusions is unlikely.

## Conclusion

The overuse of SABA remains prevalent among patients with asthma in Hong Kong despite updates in treatment recommendations to international asthma treatment guidelines. Overuse was associated with an increased risk of all-cause mortality and increased risk of hospital admissions for all-cause mortality, which was consistent with findings from our global SABINA studies. Effective physician and patient education and communication on the importance of potential adverse outcomes of SABA overuse and adherence to controller medications are key to improving asthma treatment.

### Electronic supplementary material

Below is the link to the electronic supplementary material.

Supplementary Material 1

## Data Availability

The datasets generated and/or analysed during the current study are not publicly available as the data custodians (the Hospital Authority of Hong Kong SAR) has not given permission for sharing due to patient confidentiality and privacy concerns, but are available from the corresponding author on reasonable request and with permission from the Hospital Authority of Hong Kong SAR.

## Abbreviations

CCI: Charlson Comorbidity Index
CDARS: Clinical Data Analysis and Reporting System
GINA: Global Initiative for Asthma
HA: Hospital Authority
ICD-9: International Classification of Diseases–9th Edition
ICS: Inhaled corticosteroid
LABA: Long-acting β2 agonists
LAMA: Long-acting muscarinic antagonists
LTRA: Leukotriene receptor antagonists
OCS: Oral corticosteroid
SABA: Short-acting β2 agonists
