# Supplementary material for: SABINA + Hong Kong: a territory wide study of prescribing trends and outcomes associated with the use of short-acting β2 agonists in the Chinese population
Source: BMC Pulm Med. 2024 May 14;24:232. doi: 10.1186/s12890-024-03038-1 (PMC11094848; doi:10.1186/s12890-024-03038-1)
Supplement: Supplementary file 1 — Supplementary Material 1 [file 12890_2024_3038_MOESM1_ESM.docx]

**List of Supplementary Tables**

**Supplementary Table 1.** Risk of all-cause mortality among SABA canister groups stratified by in-patient oral corticosteroids prescription

**Supplementary Table 2.** Frequency of hospital admissions associated with SABA use stratified by in-patient oral corticosteroids prescription

**Supplementary Table 1.** Risk of all-cause mortality among SABA canister groups stratified by in-patient oral corticosteroids (OCS) prescription

| With OCS prescription (N = 5,705) | | | | | Without OCS prescription (N = 12,077) | | | | |  |
| --- | --- | --- | --- | --- | --- | --- | --- | --- | --- | --- |
| SABA canister use | No. of patients/ no. of deaths | Crude HR (95% CI) | Adjusted HR (95% CI) | *P* values | SABA canister use | No. of patients/ no. of deaths | Crude HR (95% CI) | Adjusted HR (95% CI) | *P* values | |
| ≤2 canisters/year | 7,266/32 | 1.0 (Ref) | 1.0  (Ref) |  | ≤2 canisters/year | 7,266/202 | 1.0 (Ref) | 1.0 (Ref) |  | |
| 3-6 canisters/year | 3,276/78 | 1.17  (0.78 – 1.77) | 1.14  (0.75 – 1.73) | 0.533 | 3-6 canisters/year | 3,276/99 | 1.59  (1.25 – 2.02) | 1.25  (0.98 - 1.60) | 0.08 | |
| 7-10 canisters/year | 1,846/65 | 1.47  (0.96 – 2.24) | 1.38  (0.90 – 2.13) | 0.144 | 7-10 canisters/year | 1,846/57 | 1.91  (1.42 - 2.56) | 1.39  (1.02 – 1.89) | 0.037* | |
| ≥11 canisters/year | 5,394/599 | 3.57  (2.50 – 5.10) | 1.86  (1.28 – 2.71) | 0.001*** | ≥11 canisters/year | 5,394/232 | 4.14  (3.43 - 5.01) | 1.58  (1.26 – 1.98) | 0.001** | |

*Adjusted for age, sex, Charlson Comorbidity Index, ICS dose

**Supplementary Table 2.** Frequency of hospital admissions associated with SABA use stratified by in-patient oral corticosteroids (OCS) prescription

| With OCS prescription (N = 5,705) | | | | | Without OCS prescription (N = 12,077) | | | | |
| --- | --- | --- | --- | --- | --- | --- | --- | --- | --- |
| SABA canister use | No. of patients/ no. of admissions | Crude RR (95% CI) | Adjusted RR  (95% CI) | *P* values | SABA canister use | No. of patients/ no. of admissions | Crude RR (95% CI) | Adjusted RR (95% CI) | *P* values |
| ≤2  canisters/year | 7,266/396 | 1.0 (Ref) | 1.0  (Ref) |  | ≤2 canisters/year | 7,266/1,844 | 1.0 (Ref) | 1.0 (Ref) |  |
| 3-6  canisters/year | 3,276/747 | 0.89  (0.54 – 1.43) | 1.62  (1.03 – 2.55) | 0.038* | 3-6 canisters/year | 3,276/1,050 | 1.77  (1.24 – 2.61) | 3.21  (2.29 – 4.54) | <0.001*** |
| 7-10 canisters/year | 1,846/748 | 1.25  (0.74 – 2.07) | 7.40  (4.55 – 12.04) | <0.001*** | 7-10 canisters/year | 1,846/619 | 2.16  (1.35 – 3.66) | 1.78  (1.15 – 2.80) | 0.01* |
| ≥11  canisters/year | 5,394/6,241 | 2.56  (1.63 – 3.84) | 3.99  (2.57 – 6.19) | <0.001*** | ≥11 canisters/year | 5,394/2,212 | 3.79  (2.64 – 5.56) | 3.02  (2.11 – 4.38) | <0.001*** |
